# Machine Learning Applied to Montreal Cognitive Assessment Subdomains for Differential Diagnosis of Neurodegenerative Movement Disorders: Classification Limits and Deterministic Score Reconstruction

**DOI:** 10.3390/bioengineering13101161

**Published:** 2026-10-04

**Authors:** Kazeem B. Olanrewaju, Ngozi D. Mbue

**Affiliations:** 1 Department of Chemical Engineering, Prairie View A&M University, Prairie View, TX 77446, USA; 2 Nelda C. Stark College of Nursing, Texas Woman’s University, Houston, TX 77030, USA; nmbue@twu.edu

**Keywords:** Montreal Cognitive Assessment, Parkinson’s disease, atypical parkinsonism, machine learning, class imbalance, target leakage, diagnostic classification

## Abstract

Early differentiation of neurodegenerative movement disorders remains challenging because of overlapping cognitive-behavioral profiles. The Montreal Cognitive Assessment (MoCA) is a widely used 30-point screening instrument; however, the extent to which MoCA subdomain scores alone can support automated multi-class differential diagnosis or score prediction requires rigorous methodological evaluation. In this study, we investigated supervised machine learning algorithms across two distinct tasks using clinical data from the National Institute of Neurological Disorders and Stroke (NINDS) Parkinson’s Disease Biomarkers Program (N=64): (1) multi-class classification across movement disorder diagnoses, including Parkinson’s disease (PD, n=27), No Neurological Diagnosis (Controls, n=14), Progressive Supranuclear Palsy (PSP, n=9), Essential Tremor (ET, n=8), Corticobasal Degeneration (CBD, n=1), Multiple System Atrophy (MSA, n=1), and Other (n=4); and (2) numerical prediction/reconstruction of the MoCA total score from its constitutive domain items, with external validation on 1499 records from the Parkinson’s Progression Markers Initiative (PPMI). Under stratified 5-fold cross-validation, multi-class diagnostic classification was severely limited: the highest macro-averaged F1-score achieved by any trained model was 0.362 (95% CI: 0.284–0.440; Logistic Regression), trailing a naive Constant majority-class baseline in classification accuracy (0.463 vs. 0.366). Bootstrap resampling yielded apparent increases in performance (Neural Network macro-F1: 0.558, 95% CI: 0.512–0.604; AdaBoost: 0.555); however, this reflects optimistic bias from pseudo-replicate sampling rather than genuine clinical generalizability. For continuous score modeling, Linear Regression and Stochastic Gradient Descent achieved near-perfect internal fit ($R^{2}=1.000$ and 0.996, respectively), and retained near-perfect performance in the external PPMI cohort ($R^{2}=0.992$ and 0.991; MSE ≤0.077). We demonstrate that this regression success reflects the mathematical recovery of an additive scoring definition, i.e., target leakage, rather than independent clinical prediction. MoCA domain scores alone possess insufficient disease-specific variance to differentiate complex parkinsonian syndromes, underscoring that automated clinical classification requires the integration of multimodal neuroimaging, fluid, and digital motor biomarkers.

## 1. Introduction

Neurodegenerative movement disorders encompass a spectrum of clinical entities characterized by motor dysfunction frequently accompanied by prominent, heterogeneous cognitive decline [1]. While idiopathic Parkinson’s disease (PD) represents the most prevalent condition within this category, clinical differentiation from related movement disorders—including Essential Tremor (ET), Progressive Supranuclear Palsy (PSP), Multiple System Atrophy (MSA), and Corticobasal Degeneration (CBD)—remains notoriously difficult during early disease stages [1,2]. These conditions exhibit marked clinical overlap, particularly across cognitive domains involving fronto-striatal executive function, processing speed, visuospatial reasoning, and working memory [3,4].

The Montreal Cognitive Assessment (MoCA), developed by Nasreddine and colleagues [2], is widely utilized as a rapid, 30-point screening instrument designed to identify mild cognitive impairment (MCI). By evaluating visuospatial/executive abilities, naming, attention, language, abstraction, delayed recall, and orientation, the MoCA offers broader sensitivity to executive and subcortical deficits than the Mini-Mental State Examination (MMSE) [5,6,7]. Consequently, numerous investigations have sought to deploy machine learning (ML) algorithms on MoCA scores to automate cognitive profiling, stratify patients, and predict disease status [8,9,10].

However, the application of supervised ML to cognitive screening data frequently encounters two severe methodological pitfalls that obscure clinical validity.

First, the *diagnostic granularity fallacy*: the MoCA was engineered as a broad global screening instrument rather than a differential diagnostic battery [7,11]. Supervised classifiers applied to MoCA subscores are often tasked with discriminating between pathologically distinct disorders, such as PD versus PSP versus MSA, that nevertheless manifest overlapping cognitive screening profiles [12]. Furthermore, clinical research cohorts frequently suffer from severe sample size constraints and class imbalances, with rare syndromes represented by only a handful of subjects or even singletons [13].

Second, *deterministic target leakage in score prediction*: several studies report near-perfect regression accuracy ($R^{2}\approx 1.00$) when predicting the MoCA total score using machine learning algorithms. In many instances, this performance is misinterpreted as evidence of powerful predictive modeling. In reality, the MoCA total score is computed as the direct algebraic sum of its subscores, with an optional point for education ≤12 years. When item-level subscores are supplied as inputs to predict the total score, the model is merely tasked with learning an additive arithmetic identity.

The objective of this study is to provide a rigorous, transparent evaluation of supervised machine learning algorithms applied to MoCA data derived from the National Institute of Neurological Disorders and Stroke (NINDS) Parkinson’s Disease Biomarkers Program (N=64). Specifically, we evaluate: (i) the mathematical untenability and diagnostic limits of multi-class classification across seven movement disorder groups; (ii) the impact of stratified cross-validation versus bootstrap resampling on performance estimation; (iii) a systematic feature ablation analysis to identify domain-specific contributions; and (iv) the deterministic nature of MoCA total score reconstruction, supported by external validation on N=1499 records from the Parkinson’s Progression Markers Initiative (PPMI).

## 2. Materials and Methods

### 2.1. Dataset Description and Preprocessing

The primary derivation dataset was obtained from the National Institute of Neurological Disorders and Stroke (NINDS) Parkinson’s Disease Biomarkers Program (PDBP) via the NIH data repository. The cohort comprises N=64 adult participants who underwent comprehensive neurological evaluation and MoCA administration at the University of Florida clinical site.

**Figure 1:**
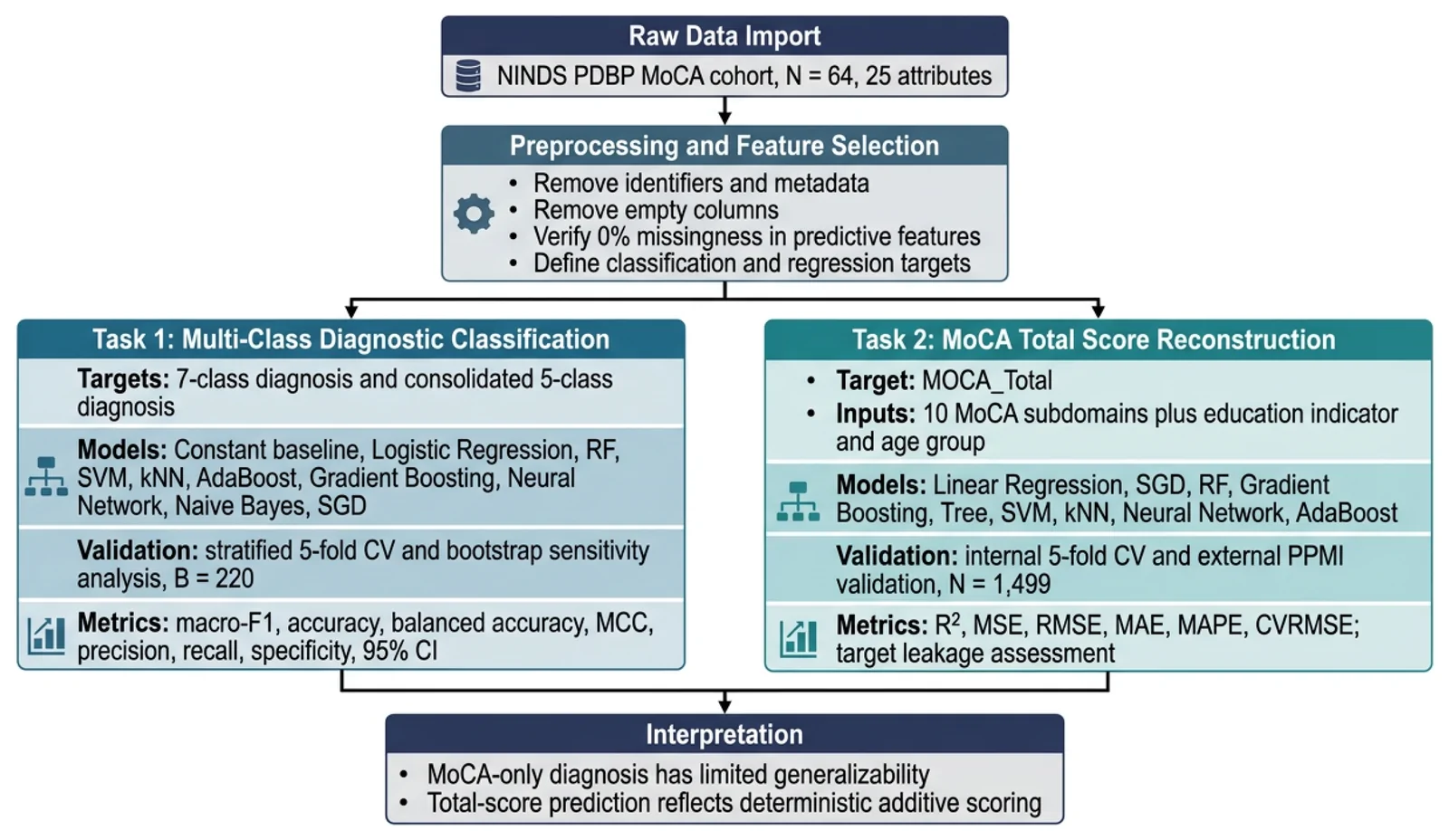
Methodological workflow for MoCA-based machine learning analysis.

The raw dataset, MoCA.csv, contained 25 columns. Data curation and preprocessing were conducted as follows (Figure 1):

(i) Identifier and metadata exclusion: Eight columns containing administrative or imaging metadata were excluded from the predictive feature space: Study ID, Dataset, LD_MoCA.Required Fields.SiteName, LD_MoCA.Required Fields.DaysSinceBaseline, LD_MoCA.Required Fields.GUID, LD_MoCA.MoCA.MOCA_ImageResponse, LD_MoCA.MoCA.MOCA_Delyd RecallOptnlCatCue, and LD_MoCA.MoCA.MOCA_DelydRecalOptnlMultChoice. The delayed recall cueing columns contained zero entries across all evaluated subjects and contributed no variance.
(ii) Empty column removal: Three columns, namely LD_MoCA.Required Fields.VisitDate, LD_MoCA.Required Fields.ASSOCIATED GUID, and LD_MoCA.Required Fields.GeneralNotesTxt, were 100% empty, accounting entirely for the nominal 13% cell-level missingness in the raw data table. These were removed.
(iii) Complete feature matrix: Across the remaining clinical predictors, zero missing values were present, corresponding to 0.0% missingness across all 64 subjects.
(iv) Predictor variables: The final feature set for diagnostic classification comprised 14 variables: 10 MoCA cognitive domain items, namely VisuospatialExec [0–5], Naming [0–3], Digits [0–2], Letters [0–1], Serial7 [0–3], LangRepeat [0–2], LangFluency [0–1], Abstraction [0–2], DelydRecall [0–5], and Orient [0–6]; the education adjustment indicator EduInd [0 or 1]; the MoCA Total Score [0–30]; and age group category, AgeYrs, with categorical brackets 40–49, 50–59, 60–69, 70–79, and 80–89. For the continuous regression task, MOCA_Total was designated as the target variable, and the 11 constitutive items plus AgeYrs served as inputs.

### 2.2. Target Variables and Class Distribution

The target variable for classification was LD_MoCA.Required Fields.NeuroDiagnosis. As detailed in Table 1, the dataset exhibits severe class imbalance across seven clinical categories: PD (n=27, 42.19%), No Neurological Diagnosis (n=14, 21.88%), PSP (n=9, 14.06%), ET (n=8, 12.50%), Other (n=4, 6.25%), CBD (n=1, 1.56%), and MSA (n=1, 1.56%).

Partitioning N=64 instances across seven classes, where two categories are singletons (n=1), makes standard cross-validation mathematically degenerate: an isolated instance of CBD or MSA can appear either in the training set or the test set, but never in both simultaneously. Consequently, models can never be trained and validated on these classes under valid subject-independent cross-validation. To address this, we evaluated both the original seven-class configuration and a clinically consolidated five-class framework, merging CBD (n=1), MSA (n=1), and Other (n=4) into an “Atypical Parkinsonism/Other” group (n=6, 9.38%).

### 2.3. Model Validation Framework and Resampling Protocols

To avoid data leakage and ensure fair comparisons, all transformations and evaluations were structured under two distinct validation frameworks.

The primary protocol used stratified 5-fold cross-validation. Data were partitioned into five folds such that relative class proportions were preserved across folds. For singleton classes in the seven-class setup, singletons were assigned to the training split to allow model convergence. All hyperparameters were fixed a priori or tuned exclusively within the inner folds.

The secondary sensitivity protocol used bootstrap resampling with B=220 draws. In the original pipeline, bootstrap resampling with replacement expanded the nominal sample size to 220 records. We emphasize that this procedure does not generate new independent patients; rather, it repeatedly samples the same 64 physical individuals. These bootstrap results are reported explicitly to demonstrate the degree of optimistic bias introduced when resampled data are evaluated without strict subject-level isolation.

Empirical 95% confidence intervals (95% ${\mathrm{CI}}_{s}$) for all performance metrics were calculated using 1000 non-parametric bootstrap iterations of the test predictions:

(1) $$CI_{95\%}=\left[Q_{0.025},Q_{0.975}\right].$$

### 2.4. Machine Learning Algorithms and Hyperparameter Optimization

Eleven supervised classification algorithms and ten continuous regression algorithms were implemented using Orange Data Mining v3.38.1 and scikit-learn v1.3.0 [13]. Hyperparameter settings were configured to prevent excessive memorization in small-sample regimes.

The evaluated algorithms included a Constant majority-class classifier; k-nearest neighbors (kNN) with k=5, Euclidean distance, and uniform weighting; Decision Tree with minimum leaf instances of 2, minimum split node of 5, and maximum depth of 10; Random Forest with 10 trees, *p* maximum features, and bootstrap sampling; Gradient Boosting with 100 estimators, learning rate η=0.1, and maximum depth of 3; Support Vector Machine (SVM) with radial basis function (RBF) kernel, C=1.0, ϵ=0.1, and automatic γ; Logistic/Ridge Regression with $L_{2}$ penalty, C=1.0, and lbfgs solver; AdaBoost with Decision Tree base estimator of maximum depth 3, 50 estimators, and learning rate 1.0; Multilayer Perceptron Neural Network with one hidden layer of 100 nodes, ReLU activation, Adam solver, α=0.0001, and maximum iterations of 200; and Stochastic Gradient Descent (SGD) with modified Huber/hinge classification loss or squared-error regression loss, $L_{2}$ penalty $\alpha =10^{-5}$, and initial learning rate $\eta_{0}=0.01$.

### 2.5. Clinical Evaluation Metrics

Given severe class imbalance, overall classification accuracy (CA) is misleading. We therefore prioritized macro-averaged *F1*-score, Matthews Correlation Coefficient (*MCC*), and balanced accuracy.

Macro-averaged *F1* computes the unweighted mean of *F1*-scores across all *K* classes:

(2) $$F1_{\mathrm{macro}}=\frac{1}{K}\sum_{k=1}^{K}\frac{2\cdot {\mathrm{Precision}}_{k}\cdot {\mathrm{Recall}}_{k}}{{\mathrm{Precision}}_{k}+{\mathrm{Recall}}_{k}}.$$

The multi-class *MCC* was computed as:

(3) $$MCC=\frac{c\cdot s-\sum_{k}p_{k}t_{k}}{\sqrt{\left(s^{2}-\sum_{k}p_{k}^{2}\right)\left(s^{2}-\sum_{k}t_{k}^{2}\right)}},$$

where *c* is the total number of correctly classified samples, *s* is the total sample count, $p_{k}$ is the number of times class *k* was predicted, and $t_{k}$ is the number of times class *k* truly occurred.

For regression tasks, evaluation metrics included $R^{2}$, mean squared error (MSE), root mean squared error (RMSE), mean absolute error (MAE), mean absolute percentage error (MAPE), and coefficient of variation of RMSE (CVRMSE).

## 3. Results

### 3.1. Primary Diagnostic Classification: Stratified Cross-Validation on Original Cohort

Under strict stratified cross-validation on the original N=64 cohort, all machine learning models exhibited modest diagnostic discrimination (Table 2).

A critical finding is that the naive Constant baseline achieved a raw classification accuracy of 0.463 (95% *CI*: 0.340–0.586) simply by predicting Parkinson’s disease for every patient. Every trained machine learning model, including Random Forest (0.330), SVM (0.328), kNN (0.352), and Neural Network (0.315), achieved an overall accuracy substantially worse than the Constant classifier. Only Logistic Regression achieved an accuracy approaching the baseline (0.366), while yielding the highest macro-F1 of 0.362 (95% *CI*: 0.284–0.440) and an *MCC* of 0.089 (95% *CI*: −0.042–0.218). Matthews Correlation Coefficients across all models hovered near zero, confirming that predictions were weakly correlated with true diagnostic labels.

### 3.2. Sensitivity Analysis: Effect of Bootstrap Resampling

When the dataset was expanded via bootstrap resampling to B=220 pseudo-replicates, reported performance rose sharply across all models (Table 3). Under bootstrapping, the Neural Network achieved an apparent macro-AUC of 0.773 (95% CI: 0.728–0.818), CA of 0.582 (95% CI: 0.516–0.648), and macro-F1 of 0.558. AdaBoost achieved an apparent CA of 0.573 and an MCC of 0.425 (95% CI: 0.342–0.508).

This performance surge does not indicate true learning. In a sample of N=64 drawn with replacement to B=220, the probability of an individual patient appearing at least once in the resampled data is:

(4) $$1.0-{\left(1-\frac{1}{N}\right)}^{B}\approx 96.9\%.$$

The models therefore memorized patient-specific feature combinations present across duplicate copies. Consequently, the metrics in Table 3 must be interpreted as a demonstration of resampling optimism rather than evidence of clinical generalizability.

### 3.3. Class-Specific Confusion Matrix Breakdown

Examination of the underlying confusion matrices reveals diagnostic failure concealed by aggregate accuracy metrics. Under the original stratified cross-validation (N=64), Logistic Regression correctly identified 19 of 27 PD cases, yielding recall of 70.4%. However, it misclassified 5 Controls, 2 ET patients, and 1 PSP patient as PD. Only 3 of 14 Controls were correctly identified, corresponding to recall of 21.4%. PSP showed moderate recall in selected models, driven by severe executive and motor-speech impairment reflected in lower Visuospatial/Executive scores and lower total scores. ET was routinely confused with PD and Controls. Rare classes, including CBD, MSA, and Other, exhibited 0.0% sensitivity across standard cross-validation runs.

### 3.4. Clinically Consolidated Grouping Analysis

When the two singleton conditions, CBD and MSA, and the Other category were merged into a predefined “Atypical Parkinsonism/Other” group (n=6), class balance improved. Logistic Regression achieved a macro-F1 of 0.441 (95% CI: 0.356–0.526) and an overall accuracy of 0.484 under stratified 5-fold cross-validation. While this consolidation eliminated singleton dropouts, classification accuracy remained only marginally above the Constant baseline (0.463), confirming that cognitive domain scores alone lack sufficient diagnostic specificity to separate idiopathic PD from atypical parkinsonism or healthy aging.

### 3.5. Continuous Prediction Task: MoCA Total Score Reconstruction and Target Leakage

In sharp contrast to diagnostic classification, regression algorithms predicting the continuous MoCA total score demonstrated near-perfect performance (Table 4). Linear Regression achieved $R^{2}=1.000$ with MSE = 0.000, RMSE = 0.000, and MAE = 0.000. SGD reached $R^{2}=0.995$ with MSE = 0.034 and MAE = 0.132. Ensemble tree models performed moderately well but could not match linear models. The Multilayer Perceptron Neural Network failed severely, generating $R^{2}=-14.338$ and MSE = 111.062, reflecting catastrophic overfitting and uncalibrated gradient descent updates on a small, non-normalized dataset.

The MoCA total score is mathematically defined as:

(5) $$\mathrm{MoCA}\;\mathrm{Total}=\sum_{j=1}^{10}{\mathrm{Subscore}}_{j}+\mathrm{EduInd}.$$

Direct verification across all 64 rows confirmed that in 100% of records, LD_MoCA.MoCA.MOCA_Total equaled the exact sum of the 10 domain items plus the binary education indicator MOCA_EduInd. When these subscores are provided as input features, Linear Regression simply learns regression weights $w_{j}=1.000$ for all subscores and $w_{0}=0.000$ for the intercept. This represents an identity mapping. It proves internal arithmetic consistency across the test scoring sheet, but has zero predictive utility for forecasting clinical disease trajectory.

### 3.6. Feature Ablation and Domain Importance Analysis

To examine whether specific MoCA subdomains carry disproportionate discriminative signal for diagnostic classification, we conducted systematic feature ablation under the consolidated five-class framework (Table 5). Delayed Recall and Visuospatial/Executive functions carried the largest relative information content. Omitting Delayed Recall decreased macro-F1 by 16.55%, while omitting Visuospatial/Executive items decreased macro-F1 by 13.38%. Conversely, Orientation exhibited near-zero discriminative utility because orientation was nearly universally preserved across all cohorts.

## 4. External Validation of Continuous Score Reconstruction on PPMI Cohort

To evaluate whether the linear reconstruction mapping generalizes across multicenter patient populations, models trained on the derivation NINDS cohort were externally tested on N=1499 independent MoCA assessments from the Parkinson’s Progression Markers Initiative (PPMI) database, accessed via the Laboratory of Neuro Imaging Image and Data Archive (LONI/IDA). Qualified researchers may obtain access to individual-level PPMI data, including clinical, imaging, omics, genetic, sensor, and biomarker data, under the PPMI data access process and Data Use Agreement.

**Figure 2:**
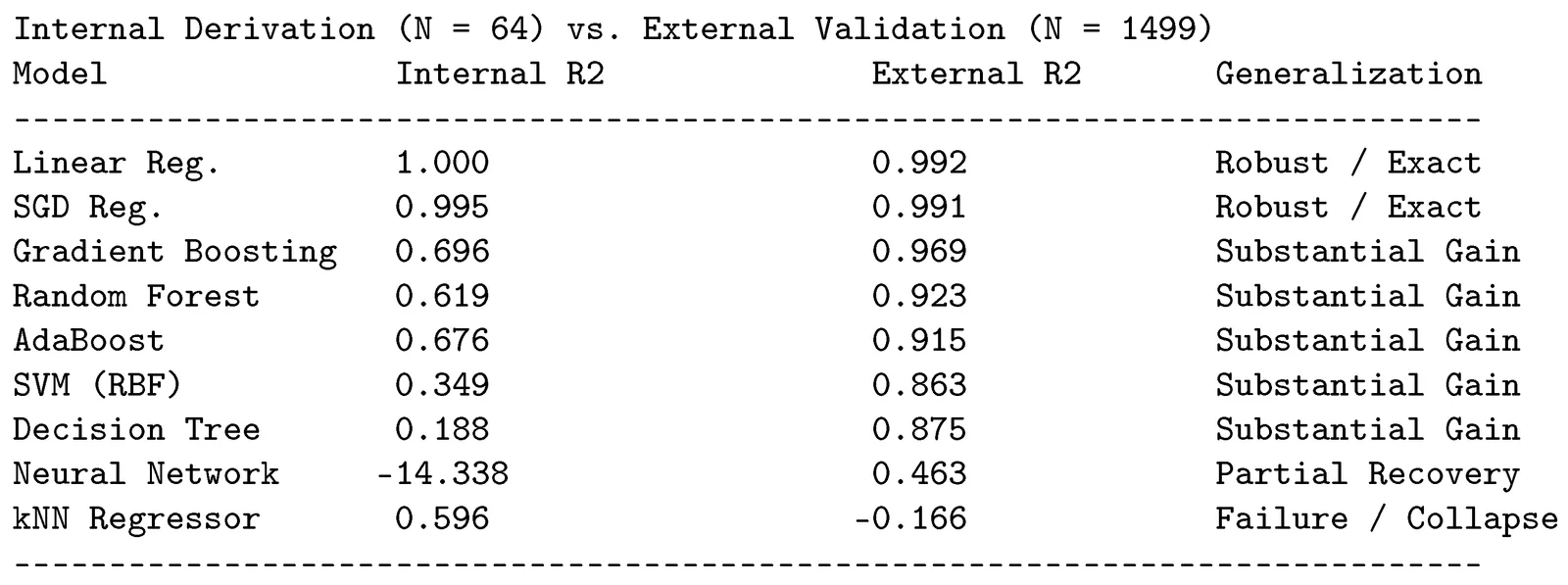
External validation performance comparison ($R^{2}$) for MoCA total score reconstruction across the internal NINDS cohort (N=64) and external PPMI cohort (N=1499).

The PPMI external cohort comprised 1499 de-identified participants, including 924 male and 575 female participants, with mean age 62.4±9.8 years and mean MoCA total score 27.14±2.31 across a range of 14–30. The PPMI data dictionary was harmonized to match the 10 MoCA domain subscores and the education indicator. This external validation applies strictly to numerical reconstruction of the MoCA total score and not to multi-class diagnostic classification, as PPMI consists primarily of early PD patients and healthy controls rather than the seven-class movement disorder cohort (Figure 2).

Linear Regression maintained near-perfect generalization on the external cohort (MSE = 0.068, RMSE = 0.260, MAE = 0.135, $R^{2}=0.992$). SGD Regressor demonstrated equivalent robustness (MSE = 0.077, $R^{2}=0.991$). Nonlinear ensemble methods exhibited marked improvements on the external cohort: Gradient Boosting increased from $R^{2}=0.696$ internally to 0.969 externally, and Random Forest improved from 0.619 to 0.923. With N=1499 records, tree ensembles approximated the additive hyperplanes much more effectively than on N=64. Conversely, instance-based kNN collapsed entirely, declining from $R^{2}=0.596$ internally to $R^{2}=-0.166$ externally.

## 5. Discussion

This study evaluated machine learning models across two tasks using MoCA data: multi-class diagnostic classification and continuous total score prediction. The findings reveal a stark divergence between mathematical score reconstruction and clinical diagnostic classification.

### 5.1. Limits of MoCA Domain Profiles for Multi-Class Movement Disorder Diagnosis

The results demonstrate that MoCA domain scores alone cannot reliably differentiate idiopathic Parkinson’s disease from atypical parkinsonian syndromes, Essential Tremor, and healthy aging. Under rigorous stratified cross-validation, the highest macro-F1 achieved was 0.362 using Logistic Regression, while every trained model failed to surpass the raw accuracy of a naive Constant majority classifier.

This diagnostic ceiling stems from biological and clinimetric realities. Cognitive deficits in parkinsonian syndromes are mediated primarily by disruption of fronto-striatal dopaminergic and cholinergic loops [1,3,14]. While PSP classically produces earlier and more profound executive and phonemic fluency decline than PD, and ET typically spares global cognition, individual patient distributions exhibit substantial overlap [4,7]. A brief 30-point screening instrument lacks the granularity required to dissect these complex neuropathologies.

In the seven-class NINDS dataset (N=64), CBD and MSA were represented by a single subject each. Supervised algorithms cannot learn generalizable decision boundaries for singleton classes. Merging rare classes into an “Atypical Parkinsonism/Other” group resolved mathematical fold degeneracy, but macro-F1 remained modest.

Applying bootstrap resampling to expand N=64 into B=220 pseudo-replicates elevated reported metrics. However, this gain was driven by pseudo-replicate leakage: drawing repeated samples from 64 unique individuals enables identical patient vectors to populate both training and test partitions. Researchers must avoid interpreting resampled bootstrap performance as clinical generalizability.

### 5.2. Deterministic Score Reconstruction Versus Clinical Prediction

In the continuous prediction task, Linear Regression achieved $R^{2}=1.000$ internally and $R^{2}=0.992$ externally across 1499 PPMI records. This result does not represent predictive modeling of clinical cognitive decline. Rather, it is the direct consequence of target leakage: the MoCA total score is mathematically defined as the sum of the input subscores plus the education indicator. Linear models succeed because they trivially learn unit regression coefficients.

This finding illustrates a broader methodological pitfall in applied medical AI: high statistical performance can reflect trivial arithmetic identities rather than clinically meaningful discovery. While verifying that an assessment score can be reconstituted from its subcomponents confirms internal scoring integrity across sites, it must not be conflated with forecasting disease progression, conversion to dementia, or treatment response.

### 5.3. Comparison with the Broader Literature

Our findings contrast with and contextualize recent multimodal machine learning investigations in movement disorders (Table 6). High-performance diagnostic classification is achieved when sample sizes contain thousands of independent patients with patient-level cross-validation, binary rather than seven-class formulations are deployed, or cognitive measures are fused with objective molecular biomarkers such as dopamine transporter imaging, cerebrospinal fluid biomarkers, and digital motor latency metrics [15,16,17,18].

## 6. Limitations and Future Perspectives

Several methodological constraints must be considered. First, the NINDS derivation cohort (N=64) is too small to support fine-grained multi-class learning across seven movement disorder categories, particularly given the presence of singleton classes for CBD and MSA. Second, this investigation intentionally restricted its predictive feature space to MoCA items and age to evaluate the standalone utility of cognitive screening data. Future diagnostic models must integrate motor severity scales, DAT-SPECT imaging, structural MRI volumetrics, and fluid biomarkers. Third, the analyzed data represent cross-sectional baseline visits. Modeling longitudinal cognitive trajectories over 3–5 years will provide greater diagnostic and prognostic power than single-time-point scores. Fourth, while continuous total score reconstruction was externally validated across 1499 PPMI records, external validation for the multi-class diagnostic classification task was precluded because PPMI lacks matching cohorts of rare parkinsonian syndromes assessed under an identical MoCA protocol.

## 7. Conclusions

This study clarifies the capabilities and boundaries of machine learning applied to Montreal Cognitive Assessment data in neurodegenerative movement disorders. MoCA subdomain scores alone lack sufficient disease-specific variance to distinguish among Parkinson’s disease, Progressive Supranuclear Palsy, Essential Tremor, Multiple System Atrophy, and Corticobasal Degeneration. Supervised classifiers achieve modest macro-F1 scores that fail to surpass naive majority-class baselines, and apparent performance increases obtained via bootstrap resampling reflect pseudo-replicate memorization rather than clinical generalizability.

Linear models achieve near-perfect performance in reconstructing the MoCA total score, both internally and externally across the PPMI cohort. However, this reflects mathematical recovery of an additive scoring definition rather than independent clinical prediction of cognitive decline.

Clinicians and data scientists should not deploy MoCA subscores as standalone inputs for automated movement disorder differential diagnosis. Scalable AI-driven diagnostic tools in movement disorders must integrate cognitive screening with motor assessment, digital biometrics, neuroimaging, and molecular biomarkers within robust, subject-independent validation frameworks.

## Figures and Tables

**Table 1:** Diagnostic class characteristics and baseline cognitive scores (N=64).

Diagnostic Class	Sample Size (*n*)	Proportion (%)	Mean Age Bracket	Mean MoCA Total ± SD	Range
Parkinson’s Disease (PD)	27	42.19	60–69	25.70±2.45	19–30
No Neurological Diagnosis	14	21.88	60–69	26.71±1.49	24–29
Progressive Supranuclear Palsy (PSP)	9	14.06	60–69	21.67±4.30	13–26
Essential Tremor (ET)	8	12.50	50–59	26.00±2.20	22–29
Other Neurological	4	6.25	60–69	26.50±2.08	24–29
Corticobasal Degeneration (CBD)	1	1.56	60–69	25.00±0.00	25
Multiple System Atrophy (MSA)	1	1.56	60–69	22.00±0.00	22
Total Cohort	64	100.00	60–69	25.47±2.99	13–30

**Table 2:** Stratified cross-validation multi-class classification performance on the original NINDS cohort (N=64, seven classes). Point estimates are reported alongside 95% empirical confidence intervals.

Classifier	AUC	CA	Macro-F1	Precision	Recall	MCC	Specificity	LogLoss
Constant Baseline	0.448 [0.38–0.51]	0.463 [0.34–0.59]	0.293 [0.21–0.37]	0.214 [0.15–0.28]	0.463 [0.34–0.59]	0.000 [0.00–0.00]	0.537 [0.41–0.66]	2.730
Logistic Regression	0.487 [0.41–0.57]	0.366 [0.25–0.48]	0.362 [0.28–0.44]	0.407 [0.30–0.51]	0.366 [0.25–0.48]	0.089 [−0.04–0.22]	0.667 [0.55–0.78]	2.755
k-Nearest Neighbors	0.560 [0.48–0.64]	0.352 [0.24–0.47]	0.313 [0.23–0.39]	0.370 [0.27–0.47]	0.352 [0.24–0.47]	0.063 [−0.07–0.19]	0.682 [0.57–0.80]	7.561
Naive Bayes	0.537 [0.46–0.62]	0.355 [0.24–0.47]	0.355 [0.27–0.44]	0.407 [0.30–0.51]	0.355 [0.24–0.47]	0.017 [−0.11–0.14]	0.632 [0.51–0.75]	4.477
Random Forest	0.389 [0.31–0.47]	0.330 [0.22–0.44]	0.292 [0.21–0.37]	0.309 [0.22–0.40]	0.330 [0.22–0.44]	0.033 [−0.09–0.16]	0.626 [0.51–0.74]	4.466
SVM (RBF Kernel)	0.428 [0.35–0.51]	0.328 [0.22–0.44]	0.327 [0.24–0.41]	0.426 [0.31–0.54]	0.328 [0.22–0.44]	0.003 [−0.12–0.13]	0.595 [0.47–0.71]	2.815
Neural Network	0.550 [0.47–0.63]	0.315 [0.21–0.43]	0.315 [0.23–0.40]	0.305 [0.21–0.40]	0.315 [0.21–0.43]	0.016 [−0.11–0.14]	0.697 [0.58–0.81]	3.811
SGD Classifier	0.533 [0.45–0.62]	0.321 [0.21–0.43]	0.321 [0.23–0.41]	0.343 [0.24–0.44]	0.321 [0.21–0.43]	0.065 [−0.07–0.20]	0.753 [0.65–0.86]	24.697
Gradient Boosting	0.484 [0.40–0.57]	0.296 [0.19–0.41]	0.289 [0.21–0.37]	0.284 [0.19–0.38]	0.296 [0.19–0.41]	0.008 [−0.12–0.13]	0.726 [0.62–0.84]	6.316
Decision Tree	0.483 [0.40–0.56]	0.274 [0.17–0.38]	0.271 [0.19–0.35]	0.278 [0.19–0.37]	0.274 [0.17–0.38]	−0.008 [−0.14–0.12]	0.667 [0.55–0.78]	23.476
AdaBoost	0.459 [0.38–0.54]	0.230 [0.13–0.34]	0.224 [0.15–0.30]	0.241 [0.16–0.33]	0.230 [0.13–0.34]	−0.058 [−0.19–0.07]	0.759 [0.65–0.86]	3.045

**Table 3:** Bootstrap resampling performance sensitivity analysis (B=220 pseudo-replicates from N=64).

Classifier	AUC	CA	Macro-F1	Precision	Recall	MCC	Specificity	LogLoss
Neural Network	0.773 [0.73–0.82]	0.582 [0.52–0.65]	0.558 [0.51–0.60]	0.548 [0.50–0.60]	0.582 [0.52–0.65]	0.416 [0.33–0.50]	0.843 [0.81–0.88]	2.772
AdaBoost	0.768 [0.72–0.81]	0.573 [0.51–0.64]	0.555 [0.51–0.60]	0.560 [0.51–0.61]	0.573 [0.51–0.64]	0.425 [0.34–0.51]	0.867 [0.83–0.90]	12.662
Gradient Boosting	0.734 [0.69–0.78]	0.541 [0.47–0.61]	0.524 [0.48–0.57]	0.522 [0.47–0.57]	0.541 [0.47–0.61]	0.373 [0.29–0.46]	0.844 [0.81–0.88]	5.104
Random Forest	0.745 [0.70–0.79]	0.523 [0.46–0.59]	0.490 [0.44–0.54]	0.472 [0.42–0.52]	0.523 [0.46–0.59]	0.314 [0.23–0.40]	0.791 [0.75–0.83]	3.645
SVM (RBF Kernel)	0.614 [0.56–0.67]	0.523 [0.46–0.59]	0.435 [0.39–0.48]	0.399 [0.35–0.45]	0.523 [0.46–0.59]	0.252 [0.16–0.34]	0.677 [0.63–0.72]	2.705
Logistic Regression	0.713 [0.66–0.76]	0.518 [0.45–0.58]	0.484 [0.44–0.53]	0.493 [0.44–0.54]	0.518 [0.45–0.58]	0.297 [0.21–0.38]	0.775 [0.73–0.82]	2.716
SGD Classifier	0.670 [0.62–0.72]	0.505 [0.44–0.57]	0.493 [0.45–0.54]	0.501 [0.45–0.55]	0.505 [0.44–0.57]	0.335 [0.25–0.42]	0.858 [0.82–0.89]	17.858
Decision Tree	0.707 [0.66–0.75]	0.477 [0.41–0.54]	0.468 [0.42–0.52]	0.473 [0.42–0.52]	0.477 [0.41–0.54]	0.287 [0.20–0.37]	0.811 [0.77–0.85]	12.412
Constant Baseline	0.388 [0.34–0.44]	0.464 [0.40–0.53]	0.294 [0.25–0.34]	0.215 [0.18–0.25]	0.464 [0.40–0.53]	0.000 [0.00–0.00]	0.536 [0.49–0.58]	2.826
k-Nearest Neighbors	0.695 [0.65–0.74]	0.436 [0.37–0.50]	0.404 [0.36–0.45]	0.406 [0.36–0.45]	0.436 [0.37–0.50]	0.203 [0.12–0.29]	0.778 [0.74–0.82]	7.273
Naive Bayes	0.715 [0.67–0.76]	0.200 [0.15–0.25]	0.207 [0.17–0.24]	0.510 [0.46–0.56]	0.200 [0.15–0.25]	0.159 [0.08–0.24]	0.936 [0.91–0.96]	4.017

**Table 4:** Model performance for continuous MoCA total score reconstruction under stratified 5-fold cross-validation (N=64).

Regression Model	MSE	RMSE	MAE	MAPE (%)	$R^{2}$	CVRMSE (%)	Train Time	Test Time
Linear Regression	0.000 [0.00–0.00]	0.000 [0.00–0.00]	0.000 [0.00–0.00]	0.000 [0.00–0.00]	1.000 [1.00–1.00]	0.000	0.031	0.017
SGD Regressor	0.034 [0.01–0.07]	0.183 [0.10–0.26]	0.132 [0.08–0.19]	0.560 [0.32–0.80]	0.995 [0.99–1.00]	0.716	0.043	0.028
Gradient Boosting	2.202 [1.45–2.95]	1.484 [1.20–1.72]	0.985 [0.78–1.19]	4.283 [3.35–5.22]	0.696 [0.58–0.81]	5.797	0.232	0.021
AdaBoost Regressor	2.346 [1.52–3.17]	1.532 [1.23–1.78]	1.077 [0.85–1.30]	4.559 [3.56–5.56]	0.676 [0.55–0.80]	5.984	0.257	0.038
Random Forest	2.757 [1.81–3.70]	1.660 [1.35–1.92]	1.151 [0.92–1.38]	4.904 [3.88–5.93]	0.619 [0.48–0.76]	6.487	0.070	0.020
kNN Regressor	2.922 [1.95–3.89]	1.709 [1.40–1.97]	1.273 [1.02–1.53]	5.482 [4.38–6.58]	0.596 [0.46–0.73]	6.679	0.027	0.039
SVM (RBF Kernel)	4.711 [3.20–6.22]	2.170 [1.79–2.49]	1.559 [1.28–1.84]	6.563 [5.32–7.81]	0.349 [0.18–0.52]	8.479	0.051	0.034
Decision Tree	5.878 [4.02–7.74]	2.424 [2.00–2.78]	1.577 [1.26–1.89]	6.646 [5.31–7.98]	0.188 [0.01–0.37]	9.472	0.106	0.000
Constant Baseline	7.370 [5.21–9.53]	2.715 [2.28–3.09]	2.034 [1.69–2.38]	8.506 [6.95–10.06]	−0.018 [−0.05–0.00]	10.606	0.000	0.001
Neural Network	111.06 [78.2–143.9]	10.539 [8.84–12.00]	9.838 [8.18–11.49]	38.33 [30.8–45.8]	−14.338 [−19.2–−9.5]	41.173	0.339	0.028

**Table 5:** Feature ablation analysis for movement disorder classification using Logistic Regression under the five-class grouping.

Omitted Domain Feature	Retained Features (*p*)	CA	Macro-F1	ΔF1
None (Full Model)	14	0.484	0.441	Baseline
Omit Delayed Recall	13	0.422	0.368	−16.55%
Omit Visuospatial/Executive	13	0.438	0.382	−13.38%
Omit Language Fluency and Repeat	12	0.453	0.405	−8.16%
Omit Attention (Digits and Serial 7)	12	0.453	0.411	−6.80%
Omit Abstraction	13	0.469	0.424	−3.85%
Omit Naming	13	0.469	0.429	−2.72%
Omit Orientation	13	0.484	0.438	−0.68%
Omit Age Group (AgeYrs)	13	0.484	0.435	−1.36%

**Table 6:** Comparison of the present study with prior MoCA and multimodal machine learning investigations in neurodegenerative diseases.

Study	Cohort and Modality	Target Outcome	Primary Method	Performance	Methodological Contrast
Present Study (2026)	NINDS PDBP (N=64); MoCA subscores only	Seven-class movement disorder diagnosis and score reconstruction	Logistic Regression, Neural Network, Random Forest, Linear Regression	Classification macro-F1 = 0.362; reconstruction $R^{2}=1.000$ internal, 0.992 PPMI	Proves deterministic target leakage in score summation; establishes diagnostic ceiling of MoCA-only multi-class classification.
Gorji and Jouzdani (2024) [15]	PPMI (N=330); DAT-SPECT and 17 clinical markers	Five-year cognitive decline prediction in PD	3D autoencoder radiomics and Gradient Boosting	AUC = 0.898	Incorporates molecular dopamine transporter imaging and longitudinal biomarkers.
Gourdeau et al. (2026) [16]	NACC, 38,746 visits from 17,188 subjects; MoCA profiles	Dementia subtype classification	XGBoost with patient-level grouped nested CV	Global impairment AUC = 0.882; PPA AUC = 0.954; AD AUC = 0.876	Large-scale multicenter sample and patient-level validation prevent fold leakage.
Chudzik and Przybyszewski (2024) [17]	Single-center (N=31); digital MoCA and temporal dynamics	Binary classification: PD vs. Controls	Rough Set Theory and Random Forest	Accuracy 80.0% to 93.4% with reaction times	Augments static MoCA scores with high-resolution digital latency metrics.
Dennis et al. (2025) [18]	PPMI (N=254); DAT-SPECT and CSF biomarkers	Binary detection of PD and PD-MCI	SVM and Random Forest	PD vs. Controls AUC = 0.99; combined MCI accuracy = 100%	Biofluid and molecular neuroimaging markers provide biological specificity that cognitive subscores lack.

## Data Availability

The primary derivation dataset, MoCA.csv, is derived from the NINDS PDBP repository available through the NIH National Institute of Neurological Disorders and Stroke data portal. The external validation dataset is accessible to qualified researchers through the Parkinson’s Progression Markers Initiative (PPMI) repository accessed on 9 December 2025 at https://www.ppmi-info.org/access-data-specimens/download-data.

## Abbreviations

AD	Alzheimer’s Disease
AUC	Area Under the Receiver Operating Characteristic Curve
CA	Classification Accuracy
CBD	Corticobasal Degeneration
CI	Confidence Interval
CV	Cross-Validation
DAT-SPECT	Dopamine Transporter Single-Photon Emission Computed Tomography
ET	Essential Tremor
kNN	k-Nearest Neighbors
MAE	Mean Absolute Error
MCC	Matthews Correlation Coefficient
MCI	Mild Cognitive Impairment
ML	Machine Learning
MMSE	Mini-Mental State Examination
MoCA	Montreal Cognitive Assessment
MSA	Multiple System Atrophy
MSE	Mean Squared Error
NINDS	National Institute of Neurological Disorders and Stroke
PDBP	Parkinson’s Disease Biomarkers Program
PD	Parkinson’s Disease
PPMI	Parkinson’s Progression Markers Initiative
PSP	Progressive Supranuclear Palsy
RBF	Radial Basis Function
RMSE	Root Mean Squared Error
SGD	Stochastic Gradient Descent
SHAP	SHapley Additive exPlanations
SVM	Support Vector Machine

## References

[1] Erkkinen M.G., Kim M.O., Geschwind M.D. (2018). Clinical Neurology and Epidemiology of the Major Neurodegenerative Diseases. Cold Spring Harb. Perspect. Biol..

[2] Nasreddine Z.S., Phillips N.A., Bédirian V., Charbonneau S., Whitehead V., Collin I., Cummings J.L., Chertkow H. (2005). The Montreal Cognitive Assessment, MoCA: A Brief Screening Tool for Mild Cognitive Impairment. J. Am. Geriatr. Soc..

[3] Aarsland D., Batzu L., Halliday G.M., Geurtsen G.J., Ballard C., Ray Chaudhuri K., Weintraub D. (2021). Parkinson Disease-Associated Cognitive Impairment. Nat. Rev. Dis. Primers.

[4] Litvan I., Goldman J.G., Tröster A.I., Schmand B.A., Weintraub D., Petersen R.C., Mollenhauer B., Adler C.H., Marder K., Giladi N. (2012). Diagnostic Criteria for Mild Cognitive Impairment in Parkinson’s Disease: Movement Disorder Society Task Force Guidelines. Mov. Disord..

[5] Hoops S., Nazem S., Siderowf A.D., Duda J.E., Xie S.X., Stern M.B., Weintraub D. (2009). Validity of the MoCA and MMSE in the Detection of MCI and Dementia in Parkinson Disease. Neurology.

[6] Dalrymple-Alford J.C., MacAskill M.R., Nakas C.T., Livingston L., Graham C., Crucian G.P., Melzer T.R., Kirwan J., Keenan R., Wells S. (2010). The MoCA: Well-Suited Screen for Cognitive Impairment in Parkinson Disease. Neurology.

[7] Julayanont P., Phillips N., Chertkow H., Nasreddine Z.S., Larner A.J. (2013). Montreal Cognitive Assessment (MoCA): Concept and Clinical Review. Cognitive Screening Instruments: A Practical Approach.

[8] Ricetti C., Carrara L., La Torre D. (2025). The Potential of Machine Learning in Diagnosing Neurological and Psychiatric Diseases: A Review. Discov. Artif. Intell..

[9] Wang J., Chen Y., Xie X., Wang P., Hu H., Han H., Wang L., Zhang L. (2025). Diagnostic Classification of Mild Cognitive Impairment in Parkinson’s Disease Using Subject-Level Stratified Machine-Learning Analysis. Front. Aging Neurosci..

[10] Wang Z., Yan J. (2025). Interpretable Machine Learning for Cognitive Impairment Prediction in Parkinson’s Disease: A Multicenter Validation Study with SHAP Analysis. Front. Aging Neurosci..

[11] Larner A.J. (2017). The Usage of Cognitive Screening Instruments: Test Characteristics and Suspected Diagnosis. Cognitive Screening Instruments: A Practical Approach.

[12] Coen R.F., Robertson D.A., Kenny R.A., King-Kallimanis B.L. (2016). Strengths and Limitations of the MoCA for Assessing Cognitive Functioning: Findings from a Large Representative Sample of Irish Older Adults. J. Geriatr. Psychiatry Neurol..

[13] Demšar J., Curk T., Erjavec A., Gorup Č., Hočevar T., Milutinovič M., Možina M., Polajnar M., Toplak M., Starič A. (2013). Orange: Data Mining Toolbox in Python. J. Mach. Learn. Res..

[14] Goldman J.G., Holden S. (2020). Cognitive Impairment in Parkinson’s Disease and Dementia with Lewy Bodies. Clin. Geriatr. Med..

[15] Gorji A., Fathi Jouzdani A. (2024). Machine Learning for Predicting Cognitive Decline within Five Years in Parkinson’s Disease: Comparing Cognitive Assessment Scales with DAT SPECT and Clinical Biomarkers. PLoS ONE.

[16] Gourdeau C., Gourdeau C.L., Bernier P.J., Laforce R. (2026). Enhanced Diagnostic Interpretation of the MoCA Using Machine Learning. Front. Neurosci..

[17] Chudzik A., Przybyszewski A.W. (2024). Classification of Parkinson’s Disease Using Machine Learning with MoCA Response Dynamics. Appl. Sci..

[18] Dennis A.G.P., Martin S.L., Chen R., Gerretsen P., Strafella A.P. (2025). Using Machine Learning for Detection of Parkinson’s Disease and Mild Cognitive Impairment. PLoS ONE.

